# Contribution of smoking and alcohol consumption to income differences in life expectancy: evidence using Danish, Finnish, Norwegian and Swedish register data

**DOI:** 10.1136/jech-2018-211640

**Published:** 2019-01-23

**Authors:** Olof Östergren, Pekka Martikainen, Lasse Tarkiainen, Jon Ivar Elstad, Henrik Brønnum-Hansen

**Affiliations:** 1 Department of Public Health Sciences, Stockholm University, Stockholm, Sweden, Europe; 2 Population Research Unit, Department of Social Research, University of Helsinki, Helsinki, Finland, Europe; 3 The Max Planck Institute for Demographic Research, Germany; 4 NOVA, Centre for Welfare and Labour Research, Oslo Metropolitan University, Oslo, Norway; 5 Department of Public Health, University of Copenhagen, Copenhagen, Denmark

**Keywords:** social inequalities, registers, mortality, smoking, alcohol

## Abstract

**Background:**

Despite being comparatively egalitarian welfare states, the Nordic countries have not been successful in reducing health inequalities. Previous studies have suggested that smoking and alcohol contribute to this pattern. Few studies have focused on variations in alcohol-related and smoking-related mortality within the Nordic countries. We assess the contribution of smoking and alcohol to differences in life expectancy between countries and between income quintiles within countries.

**Methods:**

We collected data from registers in Denmark, Finland, Norway and Sweden comprising men and women aged 25–79 years during 1995–2007. Estimations of alcohol-related mortality were based on underlying and contributory causes of death on individual death certificates, and smoking-related mortality was based on an indirect method that used lung cancer mortality as an indicator for the population-level impact of smoking on mortality.

**Results:**

About 40%–70% of the between-country differences in life expectancy in the Nordic countries can be attributed to smoking and alcohol. Alcohol-related and smoking-related mortality also made substantial contributions to income differences in life expectancy within countries. The magnitude of the contributions were about 30% in Norway, Sweden and among Finnish women to around 50% among Finnish men and in Denmark.

**Conclusions:**

Smoking and alcohol consumption make substantial contributions to both between-country differences in mortality among the Nordic countries and within-country differences in mortality by income. The size of these contributions vary by country and sex.

## Background

Despite many shared characteristics in terms of history, welfare policy arrangement, economic development and culture, there are substantial differences in life expectancy between Denmark, Finland, Norway and Sweden. Moreover, these patterns differ among men and women. In 2015, life expectancies at birth were similar among men in Finland (78.7 years) and Denmark (78.8) while being higher in Norway (80.5) and Sweden (80.4). Danish women had the lowest life expectancy (82.7), whereas life expectancy was similar among women in Finland (84.4), Norway (84.2) and Sweden (84.1).^1^

There are both similarities and differences between the Nordic countries in terms of smoking and alcohol consumption. The prevalence of daily smoking has decreased in all Nordic countries since 1980 with the exception of Finnish women, among whom smoking prevalence has remained low and stable. The decrease was the most pronounced in Denmark, although from a higher level.^2^ The prevalence of self-reported heavy episodic drinking is higher among both sexes in Finland (88% among men and 55% among women) and Denmark (80% among men and 58% among women), compared with Sweden and Norway (69% among men and 40%–44% among women).^3^

Several studies have indicated that social inequalities in health and mortality in the Nordic countries are of average magnitude in international comparisons,^4–6^ and are increasing.^7^ This finding has received attention from scholars who find it surprising that the comparatively egalitarian Nordic welfare states do not have smaller health inequalities. Smoking and alcohol, among other factors, have been suggested to contribute to this pattern.^8 9^

Previous studies conducted in Finland,^10^ Denmark^11^ and Sweden^12^ have shown that alcohol-related and smoking-related mortality make substantial contributions to social inequalities in health. These studies suggest important differences between the countries. However, directly comparing the results from these studies is difficult because of differences in the chosen indicator of social position, methods, age ranges and calendar years covered.^10–12^ The life expectancy at the country level is the aggregate of mortality patterns in population subgroups, for example, social groups. Alcohol and smoking may then contribute to both income differences in life expectancy within countries and differences in life expectancy between countries.

## Aims

We aim to assess the contribution of alcohol-related and smoking-related mortality to between-country differences and within-country income differences in life expectancy among men and women in Denmark, Finland, Norway and Sweden. We estimate the contribution of alcohol to mortality by identifying underlying and contributory alcohol-related causes of death. Smoking-related mortality is estimated using an indirect method that relies on lung cancer mortality as a marker for population exposure to smoking. First, we assess alcohol-related and smoking-related mortality by gender in the four countries and how much of the between-country differences in life expectancy can be attributed to these two risk factors. Second, we assess the contribution of alcohol-related and smoking-related mortality to the difference in life expectancy between income quintiles within countries.

## Methods

We used national register data from administrative registers in Denmark, Finland, Norway and Sweden. The data covered basic demographic characteristics, cause of death and disposable household income for the complete population between the years 1995 and 2007. The Finnish data comprised an 11% sample of the total population with an 80% oversampling of deaths for the years 1996–2007. Data on causes of death were obtained from the national cause of death registries. We restricted the analysed samples to individuals aged 25–79 years. At ages below 25 years, alcohol-related and smoking-related mortality is negligible, and income information may be less meaningful for those who are still in education. Above age 80 years, income data were not available for the Finnish population due to a large proportion of individuals in out-of-home care.

Income was defined as disposable household income after taxes and transfers, collected from tax registries, equivalised to account for differences in household composition using the Oxford method^13^ and divided into quintiles within countries. The quintiles were calculated for all individuals aged 25–79 years and updated each year. Due to missing information on household composition for 1998–2003 in the Norwegian data, data on household size for these years were imputed. As a result, the Oxford method could not be used in the Norwegian data. Instead, household income was divided with the square root of the household size. We compared the two approaches for the years where data were available and found a very high correspondence, with Pearson’s r around 0.98–0.99.

Alcohol-related deaths were identified at the individual level by the underlying or contributing cause of death as reported on the death certificate. The International Classification of Disease (ICD) guidelines dictate that all reported causes of death reflect conditions that are causally related to the death. During the observation period, both ICD-9 and ICD-10 were used. The following causes of death were classified as alcohol-related in ICD-9: alcohol-related psychosis and mental disorders (291A-F, W, X), alcohol dependence (303), alcohol abuse (305A), alcohol-related nerve damage (357F), alcoholic myopathy (425F), alcoholic gastritis (535D) and alcoholic liver disease and liver cirrhosis (571A-D); in ICD-10: alcohol-related psychosis and mental disorders (F10.0–9), alcohol-related nerve damage (G31.2), alcohol-induced epilepsy (G40.5), alcoholic myopathy (G72.1), alcoholic cardiomyopathy (I42.6), alcoholic gastritis (K29.2), alcoholic liver cirrhosis (K70.0–4), alcoholic pancreatitis (K85.2, K86.0) and accidental alcohol poisoning (X45).

The proportion of smoking-related deaths was assessed using an indirect method developed by Preston *et al*.^14^ The method uses age-specific and sex-specific lung cancer death rates to trace the population-level damage from smoking and introduces a regression model that uses lung cancer mortality data from 21 high-income countries for the period 1950–2007 to predict mortality from other causes of death. The regression coefficients of this model and external information on expected lung cancer death rates among non-smokers are used to estimate the overall proportion of deaths attributable to smoking. This model, together with information on observed lung cancer mortality rates among different subpopulations in the Nordic countries, are used to estimate the proportion of deaths attributable to smoking. We used an extension developed by Martikainen *et al* 15 to include individuals under age 50 years. Lung cancer deaths were defined as deaths from malignant neoplasms of the lung, trachea or bronchus (ICD-9: 162A, C-E, W, X and in ICD-10: C33, C34.0–3, 8-9). The number of smoking-related deaths were estimated by multiplying the proportion of smoking-related deaths with the observed total number of deaths.

The two methods differed in that alcohol-related deaths were identified on a case-by-case basis and smoking-related deaths were estimated at the aggregate level. In order to calculate the proportion of deaths attributable to both alcohol and smoking, we first estimated the number of smoking-related deaths among all deaths and second, among non-alcohol-related deaths. The difference between the two estimates of smoking-related deaths then indicates the proportion of deaths attributable to the combination of alcohol and smoking.

We calculated temporary life expectancies between the ages 25 and 79 years using the method by Chiang^16^. Temporary life expectancy is the average number of years lived within a set interval, thereby introducing a theoretical maximum when no deaths are observed within the age interval (in this case 55 years). The contribution of alcohol-related and smoking-related mortality to between-country and income differences in life expectancy was calculated as the difference between life expectancies subtracting alcohol-related and smoking-related deaths and the observed life expectancy. Since Sweden had the highest overall life expectancy among the Nordic countries within the specified years, it was used as the reference for comparing the countries while the top income quintile was used as the reference for income differences.

## Results

**Figure 1:**
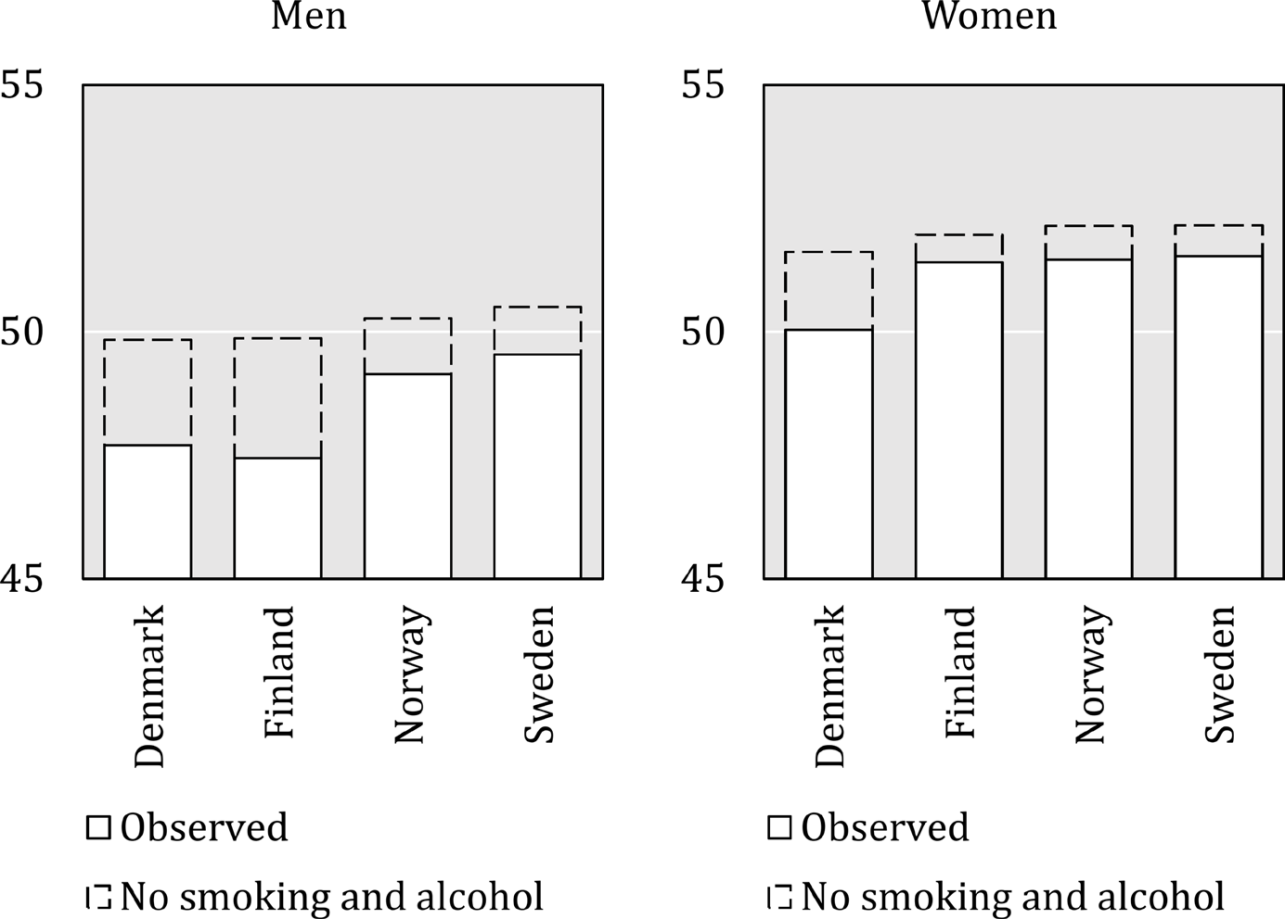
Observed temporary life expectancy between 25 and 79 years and temporary life expectancy subtracting smoking-related and alcohol-related deaths in Sweden, Norway, Finland and Denmark, men and women, 1995–2007.

Figure 1 shows observed life expectancy between the ages 25 and 79 years by sex and country. Consistent with previous findings,^1^ life expectancy was lower among men in Denmark and Finland compared with Norway and Sweden. Among women, life expectancy was similarly lower in Denmark than in the other Nordic countries while similar in Finland, Norway and Sweden.

The dashed bars in figure 1 indicate life expectancies after subtracting deaths attributable to smoking and alcohol. Among both men and women, more years of life expectancy were lost due to alcohol and smoking in the countries with lower observed life expectancy, namely Denmark and Finland. However, the majority of deaths in these ages were due to other risk factors. Exact estimates along with the contribution of each risk factor can be found in online supplementary table S2.

10.1136/jech-2018-211640.supp1 Supplementary file 1

Table 1 indicates the difference in observed life expectancy between Sweden and the other Nordic countries and the contribution of alcohol and smoking to this difference. Among men, most of the difference can be attributed to alcohol and smoking. Out of the 1.84 year difference in life expectancy between Sweden and Denmark, 1.17 years (64%) could be attributed to smoking and alcohol. Comparing Sweden and Finland, the corresponding contribution was 1.45 out of 2.09 years (70%). Although both smoking and alcohol contributed to the life expectancy disadvantage in both Denmark and Finland, alcohol was comparatively more important among Finnish men. Norwegian men had an advantage in terms of alcohol-related mortality compared with Swedish men but a disadvantage in terms of smoking-related mortality. Smoking and alcohol contributed 0.96 years (0.73+0.07+0.16) out of the 1.49 year disadvantage (64%) in life expectancy among Danish women compared with Swedish women. Smoking alone accounted for 49% (0.73 years).

**Table 1:** The contribution of alcohol and smoking to the difference in observed temporary life expectancy between Sweden and Denmark, Norway and Finland, men and women, 25–79 years, 1995–2007

		25–79 years	Difference	Contribution of risk factors (years)			
				Smoking	Smoking and alcohol*	Alcohol	Other
Men	Denmark	47.70	1.84	0.64	0.11	0.42	0.66
	Finland	47.45	2.09	0.34	0.09	1.02	0.64
	Norway	49.14	0.39	0.25	0.01	−0.10	0.23
	Sweden	49.54	Reference				
Women	Denmark	50.04	1.49	0.73	0.07	0.16	0.54
	Finland	51.40	0.13	−0.29	0.00	0.22	0.20
	Norway	51.47	0.06	0.07	0.00	−0.02	0.01
	Sweden	51.53	Reference				

Negative estimates indicate that cause-specific mortality was higher at the point of reference (Sweden).

*Contribution of deaths attributable to both smoking and alcohol.

**Figure 2:**
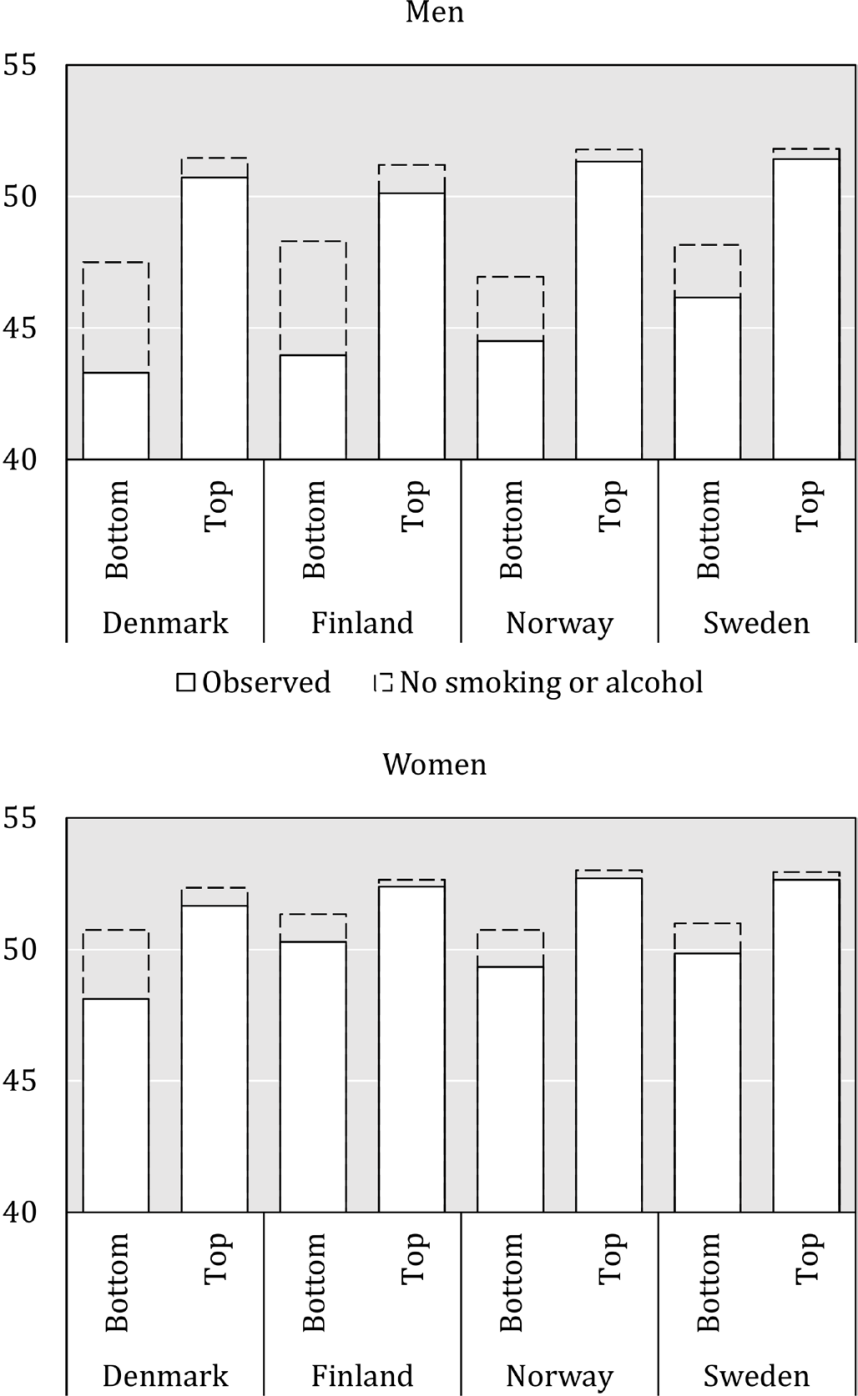
Observed temporary life expectancy and temporary life expectancy subtracting smoking-related and alcohol-related deaths in the top and bottom income quintiles, men and women in Denmark, Finland, Norway and Sweden, 25–79 years, 1995–2007.

All-cause, smoking-related and alcohol-related mortality each followed a gradient where mortality was lower the higher the income was. Here, we focus on the difference between the top and bottom quintiles. Exact estimates for each income quintile and risk factor can be found in online supplementary table S2. Figure 2 indicates observed life expectancy in the top and bottom income quintile. The dashed bars indicate life expectancies after subtracting deaths attributable to smoking and alcohol.

Table 2 indicates that among men, smoking and alcohol made the largest contributions to excess mortality by income in Denmark, where 3.44 of the 7.42 year difference (46%) in temporary life expectancy between the top and bottom income quintiles were attributable to smoking and alcohol. In Finland, the corresponding contribution was 3.28 out of 6.17 years (53%). The proportions attributable to these risk factors to income differences in life expectancy in Norway and Sweden were smaller: 29% in Norway and 31% in Sweden. Alcohol made a larger contribution in Finland and smoking made a larger contribution in Denmark.

**Table 2:** The contribution of smoking and alcohol use to differences in observed temporary life expectancy between the top and bottom income quintiles in Denmark, Finland, Norway and Sweden, men and women, 25–79 years, 1995–2007

		25–79 years		Difference	Contribution of risk factors (years)			
		Bottom	Top		Smoking	Smoking and alcohol*	Alcohol	Other
Men	Denmark	43.30	50.72	7.42	1.18	0.39	1.87	3.98
	Finland	43.96	50.13	6.17	0.77	0.28	2.23	2.89
	Norway	44.50	51.33	6.83	0.93	0.16	0.90	4.84
	Sweden	46.15	51.43	5.27	0.59	0.10	0.93	3.65
Women	Denmark	48.11	51.65	3.54	1.22	0.22	0.50	1.60
	Finland	50.29	52.39	2.10	0.18	0.05	0.57	1.30
	Norway	49.33	52.70	3.36	0.81	0.06	0.22	2.27
	Sweden	49.84	52.64	2.80	0.58	0.05	0.22	1.95

*Contribution of deaths attributable to both smoking and alcohol.

Among Danish women, 1.94 of the 3.54 year difference (55%) were attributable to alcohol and smoking; 1.22 years (34%) were attributable to smoking alone. The joint contribution of alcohol and smoking to income differences were 0.80 out of 2.10 years in Finland (38%), 1.09 out of 3.36 years in Norway (32%) and 0.85 out of 2.80 years in Sweden (30%). Alcohol made larger contributions to mortality in Finland while smoking made larger contributions in Norway and Sweden.

## Discussion

The results indicate that the majority of the difference in life expectancy between the Nordic countries can be attributed to alcohol and smoking, for both men and women. Alcohol made larger contributions to mortality among men compared with women and in Finland compared with the other Nordic countries. Smoking made comparatively larger contributions to mortality in Denmark and smaller contributions among Swedish men and Finnish women. In terms of income differences in life expectancy, the contributions of smoking and alcohol were also substantial. Nevertheless, at least half of the observed differences were due to other causes of death. This suggests that other determinants and risk factors, for example material resources, psychosocial factors and access to healthcare, are important for income differences in mortality.

## Interpretation of results

The observed patterns may partly be explained by differences in alcohol and tobacco policies. Brand *et al* 17 constructed a composite index on the strength of alcohol control policies and Joossens and Raw^18^ constructed a similar index for tobacco control. Each index consists of several dimensions of alcohol and tobacco control, including availability, pricing and advertising regulations, scoring countries on a scale between 0 and 100 (for the exact scoring criteria, see Brand *et al* 17 and Joossens and Raw^18^).

To some degree, the strength of the control policies corresponds to mortality levels. Sweden and Norway had the overall strongest alcohol and tobacco policies while Denmark had the weakest (table 3). However, Finland had stronger alcohol policies than Denmark and higher alcohol-related mortality. Policies and consumption patterns may develop partly in response to each other. A high prevalence of smoking-related and alcohol-related diseases may motivate policy changes that in turn influence consumption patterns. Furthermore, our results revealed substantial gender differences in alcohol-related and smoking-related mortality across the Nordic countries, indicating that factors other than policy influence consumption patterns since men and women share policy contexts. Patterns of alcohol and smoking depend on several interacting factors including differences in policy, culture, the distribution of economic resources, cohort effects and gender norms. Alcohol-related mortality, for example, is lower overall among women than among men within all Nordic countries and make smaller contributions to income differences in mortality among women compared with men.

**Table 3:** Summary measures of alcohol and tobacco control policies in Denmark, Finland, Norway and Sweden

	Index of alcohol control policy (Brand *et al*)	Tobacco control scale (Joossens and Raw)
Denmark	33	45
Finland	54	58
Norway	67	71
Sweden	64	60

Both indices are based on policy data from years within the range of years used in this study.

Comparing the patterns across countries and identifying departures from general patterns may give some indication of where to look for specific factors that are important within countries. For example, the comparatively low smoking-related mortality among Swedish men may be partly attributed to the use of wet smokeless tobacco, a substitute for cigarettes commonly used among Swedish men.^19^ It contains nicotine but is not a risk factor for lung cancer.^20^ The difference in life expectancy between Swedish and Danish women was largely due to smoking-attributable mortality. The prevalence of daily smokers among women in Denmark has declined, converging with the other Nordic countries.^2^ However, current smoking-related mortality is partly the result of historical smoking patterns; therefore, the high rate of smoking-related mortality among Danish women could be attributed to the high smoking prevalence of the interwar generations.^21^ Finnish men had a less dramatic social gradient in alcohol-related mortality and comparatively high levels of alcohol-related mortality in all income groups, which may be due to a generally higher level of alcohol consumption in Finland compared with the other Nordic countries.

Both Bambra^9^ and Mackenbach^8^ identify, among other factors, alcohol and smoking as potentially important contributors to social inequalities in health in the Nordic countries. However, neither of these accounts differentiate between the Nordic countries.^8 9^ Moreover, in international studies on social inequalities in health, the Nordic countries are often grouped together. In a bibliographic review of 54 studies that grouped countries using different typologies, Bergqvist *et al* 22 concluded that the results were mixed and inconclusive,^22^ and suggested that specific institutional and historical characteristics of countries may be used to understand international patterns in health inequalities. Our results corroborate both perspectives, showing that smoking and alcohol consumption make substantial contributions to social inequalities in health in all Nordic countries and reveal substantial variation between the Nordic countries in the range of these contributions, from around 30% to 55%. The contributions were larger in Denmark and among Finnish men, where the contribution of alcohol and smoking to the population life expectancy was larger. This implies that patterns in alcohol-related and smoking-related mortality at the population level and in population subgroups may have common determinants.

### Methodological considerations

We use administrative register data on cause of death to estimate deaths attributable to alcohol and smoking. The relationship between mortality and both smoking and alcohol consumption is complex, and the mortality risk depends on factors beyond total consumption.^23 24^ For example, individuals with low socioeconomic position have been observed to be more susceptible to damages from smoking and alcohol consumption,^25 26^ possibly due to relatively worse health overall^6^ and a higher likelihood to engage in multiple risky health behaviors^27^ (in line with this finding, the overlap in alcohol-related and smoking-related mortality was concentrated in the bottom income quintile (online supplementary table S2). As found in previous studies,^11 15^ this overlap is of limited magnitude. The results may not be solely attributable to differences in consumption patterns, which makes straightforward interpretations about these patterns difficult.

However, since we use register data instead of self-reported data, we avoid difficulties in accurately measuring the harmful effects of smoking and alcohol use due to non-participation, recall bias, preferential reporting and loss to follow-up.

Our methods of estimating deaths attributable to smoking^28^ and alcohol^29^ are broadly consistent with other indirect methods. Our findings are in line with those of a recent report published by the Nordic Council of Ministers, focusing on educational differences in mortality using different methods.^30^

We included individuals aged 25–79 years in order to ensure comparable data across the countries. Both alcohol-related and smoking-related mortality mainly affect individuals within this age range,^15^ which indicates that these risk factors may play a smaller role when the complete age range is considered.

Consumption patterns, especially in terms of smoking, differ substantially by cohort.^31 32^ However, country-level estimates on life expectancy as well as international comparative studies on social inequalities in health often present results using a period approach in which results are presented for a specific year or set of years. The primary aim of this study was to estimate the contribution of alcohol and smoking in the Nordic countries as reported in such studies. Therefore, we adopted a period approach. Further research adopting a cohort approach may be warranted.

We used high-quality register data with national coverage. There are differences between the countries in terms of what and how the data are collected as well as laws and regulations determining data access. For a comprehensive discussion on comparability issues, see the online supplementary comparability report.

10.1136/jech-2018-211640.supp2 Supplementary file 2

## Conclusions

Forty to seventy per cent of the between-country differences in life expectancies in the Nordic countries can be attributed to alcohol-related and smoking-related mortality. Alcohol-related and smoking-related mortality contribute 30%–55% of income differences in life expectancies within the Nordic countries. The findings indicate that while smoking and alcohol consumption make substantial contributions to between-country differences in life expectancies and within-country differences in income, there are substantial variations in these size of these contributions by country and sex.

**What is already known on this subject?**

- Social inequalities in health are not smaller in the Nordic countries despite extensive welfare programmes.
- Previous theoretical and empirical work has suggested that smoking and alcohol contribute to this pattern.
- However, few studies have focused on variations in these patterns between the Nordic countries.

**What this study adds?**

- Between 40% and 70% of the differences in life expectancy among the Nordic countries can be attributed to smoking and alcohol use.
- Between 30% and 55% of income differences in life expectancies within countries can be attributed to smoking and alcohol use.
- Smoking and alcohol make substantial contributions to social inequalities in health in the Nordic countries.
- The contributions were larger in Denmark and among Finnish men.
- Alcohol made a larger contribution among men compared with women.

## References

[1] Eurostat. Mortality and Life Expectancy Statistics. 2017 http://ec.europa.eu/eurostat/statistics-explained/index.php/Mortality_and_life_expectancy_statistics (accessed 26 Feb 2018).

[2] Ásgeirsdóttir TL, Gerdtham U-G Health behavior in the Nordic countries. Nordic Journal of Health Economics 2016;4:28–40. 10.5617/njhe.2717

[3] Wilsnack RW, Wilsnack SC, Kristjanson AF, et al Gender and alcohol consumption: patterns from the multinational GENACIS project. Addiction 2009;104:1487–500. 10.1111/j.1360-0443.2009.02696.x 19686518 PMC2844334

[4] Mackenbach JP, Stirbu I, Roskam AJ, et al Socioeconomic inequalities in health in 22 European countries. N Engl J Med 2008;358:2468–81. 10.1056/NEJMsa0707519 18525043

[5] Kunst AE, Bos V, Andersen O, et al Monitoring of trends in socioeconomic inequalities in mortality: Experiences from a European project. Demographic Research 2004;2:229–54.

[6] Eikemo TA, Huisman M, Bambra C, et al Health inequalities according to educational level in different welfare regimes: a comparison of 23 European countries. Sociol Health Illn 2008;30:565–82. 10.1111/j.1467-9566.2007.01073.x 18298629

[7] Mackenbach JP, Kulhánová I, Artnik B, et al Changes in mortality inequalities over two decades: register based study of European countries. BMJ 2016;353:i1732–8. 10.1136/bmj.i1732 27067249 PMC4827355

[8] Mackenbach JP The persistence of health inequalities in modern welfare states: the explanation of a paradox. Soc Sci Med 2012;75:761–9. 10.1016/j.socscimed.2012.02.031 22475407

[9] Bambra C Health inequalities and welfare state regimes: theoretical insights on a public health ’puzzle'. J Epidemiol Community Health 2011;65:740–5. 10.1136/jech.2011.136333 21690243

[10] Martikainen P, Peltonen R, Mäkelä P, et al The changing contribution of harmful consumption of alcohol and smoking to income differences in life expectancy: a population-based follow-up study in Finland from 1988 to 2007. Epidemiology 2013. 10.1097/EDE.0000000000000064 24487202

[11] Koch MB, Diderichsen F, Grønbæk M, et al What is the association of smoking and alcohol use with the increase in social inequality in mortality in Denmark? A nationwide register-based study. BMJ Open 2015;5:e006588 10.1136/bmjopen-2014-006588 PMC4431124 25967987

[12] Östergren O, Martikainen P, Lundberg O The contribution of alcohol consumption and smoking to educational inequalities in life expectancy among Swedish men and women during 1991-2008. Int J Public Health 2018;63:41–8. 10.1007/s00038-017-1029-7 28835983 PMC5766714

[13] OECD. The OECD List of Social Indicators. Paris: OECD, 1982.

[14] Preston SH, Glei DA, Wilmoth JR A new method for estimating smoking-attributable mortality in high-income countries. Int J Epidemiol 2010;39:430–8. 10.1093/ije/dyp360 20032265 PMC2915474

[15] Martikainen P, Mäkelä P, Peltonen R, et al Income differences in life expectancy: the changing contribution of harmful consumption of alcohol and smoking. Epidemiology 2014;25:182–90. 10.1097/EDE.0000000000000064 24487202

[16] Chiang CL The Life Table and Its Applications. Malabar: Robert E. Krieger Publishing Company, 1984.

[17] Brand DA, Saisana M, Rynn LA, et al Comparative analysis of alcohol control policies in 30 countries. PLoS Med 2007;4:e151 10.1371/journal.pmed.0040151 17455992 PMC1876414

[18] Joossens L, Raw M The Tobacco Control Scale: a new scale to measure country activity. Tob Control 2006;15:247–53. 10.1136/tc.2005.015347 16728757 PMC2564668

[19] Norberg M, Malmberg G, Ng N, et al Who is using snus? - Time trends, socioeconomic and geographic characteristics of snus users in the ageing Swedish population. BMC Public Health 2011;11:1 10.1186/1471-2458-11-929 22169061 PMC3267833

[20] Foulds J, Ramstrom L, Burke M, et al Effect of smokeless tobacco (snus) on smoking and public health in Sweden. Tob Control 2003;12:349–59. 10.1136/tc.12.4.349 14660766 PMC1747791

[21] Lindahl-Jacobsen R, Rau R, Jeune B, et al Rise, stagnation, and rise of Danish women’s life expectancy. Proceedings of the National Academy of Sciences 2016;113:4015–20. 10.1073/pnas.1602783113 PMC4839462 27035998

[22] Bergqvist K, Yngwe MA, Lundberg O Understanding the role of welfare state characteristics for health and inequalities - an analytical review. BMC Public Health 2013;13:1234 10.1186/1471-2458-13-1234 24369852 PMC3909317

[23] Bloomfield K, Grittner U, Kramer S, et al Social inequalities in alcohol consumption and alcohol-related problems in the study countries of the EU concerted action ’Gender, Culture and Alcohol Problems: a Multi-national Study'. Alcohol Alcohol Suppl 2006;41:i26–i36. 10.1093/alcalc/agl073 17030500

[24] Hiscock R, Bauld L, Amos A, et al Socioeconomic status and smoking: a review. Ann N Y Acad Sci 2012;1248:107–23. 10.1111/j.1749-6632.2011.06202.x 22092035

[25] Christensen HN, Diderichsen F, Hvidtfeldt UA, et al Joint Effect of Alcohol Consumption and Educational Level on Alcohol-related Medical Events. Epidemiology 2017;28:872–9. 10.1097/EDE.0000000000000718 28731961

[26] Nordahl H, Lange T, Osler M, et al Education and cause-specific mortality: the mediating role of differential exposure and vulnerability to behavioral risk factors. Epidemiology 2014;25:389–96. 10.1097/EDE.0000000000000080 24625538

[27] Laaksonen M, Prättälä R, Lahelma E Sociodemographic determinants of multiple unhealthy behaviours. Scand J Public Health 2003;31:37–43. 10.1080/14034940210133915 12623523

[28] Martikainen P, Ho JY, Preston S, et al The changing contribution of smoking to educational differences in life expectancy: indirect estimates for Finnish men and women from 1971 to 2010. J Epidemiol Community Health 2013;67:219–24. 10.1136/jech-2012-201266 23201620 PMC3886806

[29] Trias-Llimós S, Martikainen P, Mäkelä P, et al Comparison of different approaches for estimating age-specific alcohol-attributable mortality: The cases of France and Finland. PLoS One 2018;13:e0194478 10.1371/journal.pone.0194478 29566081 PMC5864025

[30] Juel K, Davidsen M, Rosendahl Jensen HA Social Inequality in Mortality in the Nordic Countries: The impact of smoking and alcohol Nordic Council of Ministers N-N. Copenhagen: Nomesko, 2017.

[31] Preston SH, Wang H Sex mortality differences in the United States: the role of cohort smoking patterns. Demography 2006;43:631–46. 10.1353/dem.2006.0037 17236538

[32] Bray FI, Weiderpass E Lung cancer mortality trends in 36 European countries: secular trends and birth cohort patterns by sex and region 1970-2007. Int J Cancer 2010;126:NA–66. 10.1002/ijc.24855 19728330

