# Supplementary material for: Contribution of smoking and alcohol consumption to income differences in life expectancy: evidence using Danish, Finnish, Norwegian and Swedish register data
Source: J Epidemiol Community Health. 2019 Jan 23;73(4):334–9. doi: 10.1136/jech-2018-211640 (PMC6581103; doi:10.1136/jech-2018-211640)
Supplement: Supplementary file 1 [file jech-2018-211640supp001.pdf]

Table S1. Observed temporary life expectancy, temporary life expectancy subtracting smoking and alcohol-related deaths and the specific contribution of the specific risk factors, men and women in Denmark, Finland, Norway and Sweden, 25—79 years, 1995—2007

		e(25—79)			Contribution of risk factors (yrs)		
		Obs.	No smo. & alc.	Diff.	Smoking	Smo. + Alc.	Alcohol
Men	Denmark	47.70	49.84	2.14	1.18	0.14	0.81
	Finland	47.45	49.86	2.42	0.88	0.12	1.41
	Norway	49.14	50.27	1.12	0.79	0.04	0.29
	Sweden	49.54	50.50	0.96	0.54	0.03	0.39
Women	Denmark	50.04	51.62	1.58	1.24	0.09	0.26
	Finland	51.40	51.96	0.56	0.22	0.02	0.32
	Norway	51.47	52.15	0.68	0.59	0.02	0.08
	Sweden	51.53	52.16	0.63	0.51	0.02	0.10

Table S2. Observed temporary life expectancy, temporary life expectancy subtracting smoking and alcohol-related deaths and the specific contribution of the specific risk factors by income quintile, men and women in Denmark, Finland, Norway and Sweden, 25—79 years, 1995—2007

			e(25—79)			Contribution of risk factors (yrs)		
		Income	Obs.	No smo. & alc.	Diff.	Smoking	Smo. + Alc.	Alcohol
Men	Denmark	1	43.30	47.50	4.20	1.72	0.41	2.07
		2	46.81	49.14	2.33	1.41	0.12	0.80
		3	48.74	50.29	1.55	1.12	0.05	0.38
		4	49.74	50.85	1.11	0.82	0.02	0.26
		5	50.72	51.47	0.75	0.53	0.01	0.21
	Finland	1	43.96	48.31	4.35	1.15	0.30	2.90
		2	45.69	48.90	3.21	1.17	0.20	1.84
		3	47.75	49.84	2.09	0.86	0.09	1.14
		4	48.92	50.49	1.57	0.66	0.05	0.86
		5	50.13	51.20	1.08	0.38	0.02	0.68
	Norway	1	44.50	46.94	2.44	1.30	0.16	0.98
		2	47.71	49.29	1.58	1.06	0.06	0.46
		3	49.78	50.68	0.90	0.70	0.02	0.18
		4	50.64	51.32	0.68	0.55	0.01	0.12
		5	51.33	51.78	0.45	0.37	0.00	0.08
	Sweden	1	46.15	48.17	2.01	0.86	0.11	1.04
		2	47.80	49.31	1.51	0.77	0.06	0.67
		3	49.76	50.65	0.89	0.54	0.02	0.32
		4	50.65	51.28	0.62	0.42	0.01	0.19
		5	51.43	51.81	0.39	0.27	0.00	0.11
Women	Denmark	1	48.11	50.74	2.63	1.80	0.23	0.60
		2	49.35	51.33	1.98	1.62	0.10	0.27
		3	50.29	51.67	1.38	1.19	0.04	0.15
		4	50.98	52.03	1.05	0.90	0.03	0.12
		5	51.65	52.34	0.69	0.57	0.01	0.10
	Finland	1	50.29	51.34	1.05	0.28	0.05	0.72
		2	50.62	51.45	0.83	0.34	0.03	0.46
		3	51.42	51.95	0.52	0.26	0.02	0.25
		4	51.95	52.31	0.36	0.16	0.01	0.19
		5	52.39	52.65	0.26	0.10	0.00	0.15
	Norway	1	49.33	50.73	1.40	1.09	0.06	0.25
		2	50.96	51.84	0.87	0.76	0.02	0.09
		3	51.94	52.50	0.55	0.49	0.01	0.06
		4	50.64	51.07	0.43	0.39	0.00	0.04
		5	52.70	53.01	0.31	0.28	0.00	0.03
	Sweden	1	49.84	50.98	1.14	0.83	0.05	0.26
		2	50.68	51.69	1.01	0.83	0.04	0.15
		3	51.74	52.34	0.60	0.51	0.01	0.08
		4	50.65	51.07	0.42	0.36	0.01	0.05
		5	52.64	52.94	0.29	0.25	0.00	0.04
