# Supplementary material for: Contribution of smoking and alcohol consumption to income differences in life expectancy: evidence using Danish, Finnish, Norwegian and Swedish register data
Source: J Epidemiol Community Health. 2019 Jan 23;73(4):334–9. doi: 10.1136/jech-2018-211640 (PMC6581103; doi:10.1136/jech-2018-211640)
Supplement: Supplementary file 2 [file jech-2018-211640supp002.pdf]

WEB SUPPLEMENT

The contribution of smoking and alcohol consumption to income differences in life expectancy - Evidence using Danish, Finnish, Norwegian and Swedish register data.

Comparability report

1. Introduction

The level of detail and high quality of administrative registers kept in the Nordic countries represent a valuable resource for comparative research. However, several potential issues that warrant consideration in order to ensure the comparability of these registers across the different countries. In this report, we first present a systematic approach to identifying comparability issues in register-based research. Second, we apply this approach to each variable included in the analysis, and provide a detailed account of issues of comparability related to the complete process of conducting the analysis. The comparability issues under consideration may be divided into two main categories; *availability* and data *content*.

Availability. The access to register data is strictly regulated in all Nordic countries; in order to gain access to data, research projects need to be vetted both from an ethical perspective and from a data protection perspective. For a summary of the ethical vetting process in the Nordic countries, see Ludvigsson et al. (2015). The specific technical solutions and practices of statistical agencies also influence the format of the data and the way in which the data are delivered to researchers.

Content. Differences in content may be divided into three related types of issue. These issues influence comparability both between countries and within countries over time. 1) *Differences in what data are collected*: The registers are kept for administrative purposes and differences in the institutional environment and political decisions impact what information is collected and stored. 2) *Differences in methods of data collection:* The specific practices surrounding data collection, reporting and validation influence the quality of the data. For example, differences in medical practices across countries may influence health data. Similarly, differences in the tax code and social services influence the reporting of socioeconomic data. 3) *Differences in definitions*: What is included in the data may differ between the countries. One type of difference relates to the classifications used, for example separate standards of classification of occupations and educational attainment. Another difference is less straightforward and relates to how specific information relates to the overall institutional and social environment in each country. Comparing specific welfare programs and subsidies may be misleading as they are structured in relation to each other and what is covered by specific programs may differ between countries. Both of these types of issues may be addressed by aggregating or reclassifying variables so that they indicate the same phenomena. This process often implies giving up some level of detail in order to ensure comparability.

2. Comparability issues of specific variables

2.1 Study population

We restricted the study population and observation period to those years for which data on demographic information, socioeconomic conditions and causes of death were available and eligible for analysis, given valid ethical permissions to the participating researchers in all four countries. The population in this study comprised all men and women aged 25—79 in Denmark, Norway and Sweden during 1995—2007 and an 11% sample of the total Finnish population with an 80% oversampling of deaths during 1996—2007. As the Finnish data do not include the total population, the estimates for Finland may be less precise. However, as shown by Martikainen et al. (2014), the confidence intervals for life expectancies are already very narrow (around +/- 0.2 years) using the same underlying dataset. This previous analysis was based on a set of five-year

follow-up periods while the current study uses 11 years of follow up, yielding more precise estimates. This suggests that the differences observed between countries and between groups within Finland are not due to random variation.

2.2 Disposable income

Register data on income are collected by the tax authority. As such, registered income and information may be lacking due to income earned abroad or tax evasion. Within countries, detailed data on the specific sources of income exist but the level of detail available to researchers varies across countries. Furthermore, differences in specific transfers and subsidies in terms of their levels and relationhips to each other change across countries and over time. Specific income sources, for example income resulting from unemployment or sick pay, may be used to compare exposure to adverse conditions across countries while summary measures, for example, labor income, capital income or disposable income, may present more comparable estimates on the economic conditions of individuals.

In this study, we used quintiles of disposable household income, equivalized for household composition. Income quintiles are a comparable measure the individuals' relative position within the country while not taking into account differences in the economic and social conditions between countries. In Finland, a large part of the population over the age of 80 live in institutions and information on income for older ages is largely missing from the register. Consequently, we excluded the population aged 80 and older in all countries.

2.3 Household composition

In order to compare the disposable income of individuals in different household compositions, income was equivalized using the Oxford method (OECD, 1982). Each household member is assigned a weight. The first adult is assigned 1 and subsequent adults are assigned 0.7 while children are assigned 0.5. A household weight is then calculated by adding the individual weights. A household of two adults and a child is then given a household weight of 2.2 (1+0.7+0.5). Finally, the disposable household income is divided by the household weight. In terms of comparability, using household income instead of individual income is an advantage since some welfare programs and transfers take resources in the household into account while others are directed at one member of the household.

In Sweden, cohabiters are identified through either marriage, registered partnership or if the couple has a child. Therefore, unmarried cohabiters without a child are classified as singles (since 2011, cohabiters without children can be identified due to the implementation of a register on dwellings). Using the Oxford method, the equivalized income of two adults without a child is calculated by dividing the total income by 1.7 (OECD, 1982). Using the individual income does not take into account the benefit of sharing housing costs and when the difference in income is larger between the partners, the difference between the equivalized and the individual income is larger.

In the Norwegian data, information on family size and family composition came from updated public registers for the years 2004—2007, but was partly lacking for the previous years, especially for the 1998—2003 period. Imputation methods were used in order to overcome this problem. Since the age of children are uncertain for the imputed values, we were unable to apply the Oxford method. Instead, the household income was equivalized by dividing the household income by the square root of the total household size. As a sensitivity analysis, the two methods were compared for the years with sufficient data finding that the income quintiles assigned by the two methods are highly correlated (0.98—0.99).

2.4 Cause of death

We used data on cause of death from the national cause of death registers in Denmark, Norway, Finland and Sweden reported using the international classification of disease (ICD), versions nine and 10. Detailed descriptions on the specific procedures of reporting causes of death into national

registers have been published elsewhere for Denmark (Helweg-Larsen, 2011), Finland (Lahti & Penttilä, 2001), Norway (Pedersen & Ellingsen, 2015) and Sweden (Brooke et al., 2017). There are differences in practice in terms of the medical investigation, reporting, validation and administration of the registers. Phillips et al. (2014) developed an index scoring cause of death registers on basis of (among other criteria) quality, consistency and coverage. Out of the Nordic countries, Finland scored 0.956 while the other Nordic countries had somewhat lower scores (Sweden 0.894, Denmark 0.878, Norway 0.876). Overall, the quality of the cause of death registers in the Nordic countries are high by international standards, and validation studies tend to conclude that they present valid estimates at population levels while the validity of individual deaths are less certain.

The method used for estimating smoking-related mortality relies on the accurate classification of lung cancer deaths. Several evaluation studies have indicated high reliability in the classification of lung cancer deaths (see for example Doria-Rose and Marcus (2009) and Johansson and Westerling (2000)). When classifying alcohol-related deaths, we used a range of different diagnoses. Since both the underlying and contributing causes of death indicate conditions that are causally related to the death, we classified a death as alcohol-related if an alcohol-related cause was recorded as either the underlying or any contributing cause of death. Studies from Japan (Mieno et al., 2016) and the US (Cheng et al., 2012) indicate that in cases where the underlying cause of death is recorded incorrectly, the true underlying cause of death is often recorded as a contributing cause of death. Using both contributing and underlying causes of death then reduces the risk of misclassification since this method is not sensitive to errors in classifying the causal sequence of the causes of death. The risk of misclassification is elevated for deaths at advanced ages due to comorbidity (Johansson & Westerling, 2000). The population in this study comprised individuals aged 25—79 for whom the risk of misclassification is lower.

Random misclassification is a greater problem for studies that rely on accurate classification of individual deaths compared to studies on population-level rates. However, previous studies have suggested an underreporting of alcohol-related causes of death on death certificates due to the social stigma of alcoholism and death certificates being public documents (Pierce & Denison, 2010). It is then possible that our estimates underestimate the contribution of alcohol to mortality and if the underreporting differs between countries and income groups, it is further possible that the contribution of alcohol to differences in mortality is inaccurate. We are unaware of any studies empirically evaluating this possibility.

This study largely avoids the main concerns involved when analyzing data from cause of death registers: the countries maintain high-quality cause of death registers. We use either causes of deaths with a low level of misclassification (lung cancer) or several different diagnoses that are not sensitive to errors in sequence. The included age range is comparatively young, which reduces the risk of misclassification due to comorbidity.

This research was conducted as part of the C-Life-project (Nordforsk Grant No. 75970). One of the aims of the C-Life project is to document the comparability between the Nordic countries when conducting research on register data linking health and social data. By publishing in-depth reports on the issues concerning the complete research process, we aim to promote knowledge of comparability issues within the research community.

3. References

Brooke, H. L., Talbäck, M., Hörnblad, J., Johansson, L. A., Ludvigsson, J. F., Druid, H., Feychting, M., & Ljung, R. (2017). The Swedish cause of death register. *European Journal of Epidemiology, 32*(9), 765-773.

Cheng, T.-J., Lin, C.-Y., Lu, T.-H., & Kawachi, I. (2012). Reporting of incorrect cause-of-death causal sequence on death certificates in the USA: using hypertension and diabetes as an educational illustration. *Postgraduate Medical Journal*, postgradmedj-2012-130912.

Doria-Rose, V. P., & Marcus, P. M. (2009). Death certificates provide an adequate source of cause of death information when evaluating lung cancer mortality: An example from the Mayo Lung Project. *Lung Cancer, 63*(2), 295-300.

Helweg-Larsen, K. (2011). The Danish register of causes of death. *Scandinavian Journal of Public Health, 39*(7_suppl), 26-29.

Johansson, L. A., & Westerling, R. (2000). Comparing Swedish hospital discharge records with death certificates: Implications for mortality statistics. *International Journal of Epidemiology, 29*(3), 495-502.

Lahti, R. A., & Penttilä, A. (2001). The validity of death certificates: Routine validation of death certification and its effects on mortality statistics. *Forensic Science International, 115*(1-2), 15-32.

Ludvigsson, J. F., Håberg, S. E., Knudsen, G. P., Lafolie, P., Zoega, H., Sarkkola, C., von Kraemer, S., Weiderpass, E., & Nørgaard, M. (2015). Ethical aspects of registry-based research in the Nordic countries. *Clinical Epidemiology, 7*, 491.

Martikainen, P., Mäkelä, P., Peltonen, R., & Myrskylä, M. (2014). Income differences in life expectancy: The changing contribution of harmful consumption of alcohol and smoking. *Epidemiology, 25*(2), 182-190.

Mieno, M. N., Tanaka, N., Arai, T., Kawahara, T., Kuchiba, A., Ishikawa, S., & Sawabe, M. (2016). Accuracy of death certificates and assessment of factors for misclassification of underlying cause of death. *Journal of Epidemiology, 26*(4), 191-198.

OECD. (1982). *The OECD List of Social Indicators*. Paris: OECD.

Pedersen, A., & Ellingsen, C. (2015). Data quality in the causes of death registry. *Tidsskrift for den Norske Laegeforening: Tidsskrift for Praktisk Medicin, Ny raekke, 135*(8), 768.

Phillips, D. E., Lozano, R., Naghavi, M., Atkinson, C., Gonzalez-Medina, D., Mikkelsen, L., Murray, C. J., & Lopez, A. D. (2014). A composite metric for assessing data on mortality and causes of death: The vital statistics performance index. *Population Health Metrics, 12*(1), 14.

Pierce, J., & Denison, A. (2010). Accuracy of Death Certifications and the Implications for Studying Disease Burdens *Handbook of Disease Burdens and Quality of Life Measures* (pp. 329-344): Springer.
